# Hierarchy or Heterarchy of Mammalian Circadian Timekeepers?

**DOI:** 10.1177/07487304241286573

**Published:** 2024-10-24

**Authors:** William Bechtel

**Affiliations:** Department of Philosophy, University of California, San Diego, La Jolla, California

**Keywords:** hierarchy, heterarchy, suprachiasmatic nucleus, peripheral clocks, zeitgebers

## Abstract

Mammalian circadian biologists commonly characterize the relation between circadian clocks as hierarchical, with the clock in the suprachiasmatic nucleus at the top of the hierarchy. The lineage of research since the suprachiasmatic nucleus (SCN) was first identified as *the clock* in mammals has challenged this perspective, revealing clocks in peripheral tissues, showing that they respond to their own zeitgebers, coordinate oscillations among themselves, and in some cases modify the behavior of the SCN. Increasingly circadian timekeepers appear to constitute a heterarchical network, with control distributed and operating along multiple pathways. One reason for the continued invocation of hierarchy in mammalian circadian biology is that it is difficult to understand how a heterarchical system could operate effectively so as to maintain the organism. Evolved mechanisms, however, need not respect hierarchy and those that have survived have demonstrated the ability of heterarchical organizaton to maintain organisms.

Because of the widespread presence of clocks throughout the body, the circadian system of an animal resembles a clock shop^1^ rather than a single clock. Therefore, the important question arises of how rhythms of so many clocks are efficiently synchronized.

—(Husse et al., 2015)

We all employ timekeeping devices—clocks, watches, cell phones, and so on. Each of these devices shares a property with biological circadian clocks—its period is only *circa* or approximately equal to the period of the earth’s rotation around the sun. In the case of circadian clocks, this means that if they are allowed to free-run, they will soon be out of sync with the day-night cycle in the environment. What prevents this is that they are regularly realigned in response to cues from the environment, known as *zeitgebers* (“*time givers*”). In the 21st century, we are less cognizant of this feature of our watches or cell phones as they have become extremely precise. But a few generations ago, people had to reset their watches or, less frequently, clocks, as they had “lost” or “gained” time. Now cell phones do this without our awareness.

One response to the need to reset timekeepers is to identify a zeitgeber for each timekeeper and reset the timekeeper in accord with that. Applied to human timekeeping, that involves each owner of a watch or clock measuring solar time (e.g. by detecting when the sun is at the zenith in their location) and adjusting their device. While this strategy may have worked when humans were largely isolated, it is not practical when they need to coordinate temporally with other human beings. Humans have adopted a hierarchical scheme. At first, a town or a company maintained its own time but, with the railroads and long-distance communication, it became important to standardize time, first within a country, and eventually across the planet. For a period, the observatory at Greenwich in England performed the official measurements. With further quests for accuracy, those responsible for timekeeping turned to atomic clocks. Even atomic clocks vary; currently, official time is maintained by a network of more than 300 atomic clocks distributed at more than 60 laboratories. The result is a hierarchy of timekeepers, with those lower in the hierarchy taking their cue from those higher. The only task for local devices—watches or cell phones—is to stay entrained to signals from on top.

Circadian researchers have assumed a similar hierarchy of timekeepers within organisms. In mammals, the supreme clock is taken to be located in the suprachiasmatic nucleus (SCN) of the hypothalamus. Using light as a zeitgeber, the SCN clock makes measurement about time in the external world and is assumed to direct activities in the rest of the organism. (Like the network of atomic clocks, the SCN relies on a network of connections between individual neuronal timekeepers.) Unlike in the history of human timekeeping, where time was first kept locally and then centralized, research on circadian timekeeping initially assumed the SCN was the only clock. Peripheral clocks were identified later, leading to the recognition that nearly every mammalian cell has the machinery to keep time. Following the early evidence, which suggested that these local timekeepers could not maintain oscillations on their own, peripheral clocks were assumed to be directed top-down by the SCN.

While circadian researchers continue to characterize the circadian system as organized hierarchically, I will argue that the trajectory of research has progressively challenged that perspective. To refer to the failure of hierarchy in human preferences (e.g. situations in which a person prefers A to B, B to C, but C to A), McCulloch (1945) introduced the term *heterarchy*. For now, it will suffice to view a heterarchical system as one lacking a top-level authority. I will return to develop a somewhat fuller account of heterarchy of control systems and how it can provide for coherent behavior in biological organisms in section “The SCN is different but not at the top.” Once one abandons hierarchy, it is natural to view the different clocks as forming a network and to focus on how each responds to its own zeitgebers but, as a result of being coupled, are tuned by others (a perspective recently articulated by Woller and Gonze, 2021). Before developing the heterarchical network perspective, I will show that the history of research on mammalian circadian timekeeping has generated a succession of findings that deviate from the hierarchical perspective.

In developing accounts of the mammalian circadian timekeeping system, circadian researchers have invoked a series of metaphors. As I describe in section “Identifying the SCN as *the Clock*,” once researchers accepted that circadian rhythms were generated endogenously, they searched for *the clock*. This quest was extremely productive, not only revealing the SCN as a timekeeper but enabling research on how it keeps time. This facilitated the discovery of peripheral clocks. Because the research I discuss in section “Clockworks but not autonomous clocks in every tissue” seemed to show that these peripheral clocks only kept time when they are directed by the SCN, the peripheral clocks were often viewed as subordinate or derivative.^2^ In section “Endogenous clocks in peripheral tissues that require the SCN for entrainment,” I relate how, as a result of research revealing that individual fibroblasts maintain oscillations but lose synchrony without input from the SCN, the SCN came to be viewed as an orchestra conductor and the peripheral clocks as musicians who know how to play their instruments but require coordination.

One view of musicians in an orchestra is that they perform just as directed by the conductor. This is a hierarchical conception—control stems from the conductor or the SCN. But, as I describe in section “Alternative entrainment for endogenous clocks,” early in this century researchers began to find evidence that the peripheral clocks could take their own measurements of time in the external world—they responded to different zeitgebers than the SCN. They might still be characterized as musicians, but as ones who could develop their own ideas about how to play. From this perspective, each peripheral clock is individually situated between input from the SCN and its own zeitgeber. An important factor in enabling local components to resist a higher authority is whether they can coordinate with each other. As I discuss in section “Peripheral clocks can coordinate themselves without the SCN,” evidence is growing of communication, both between clocks within an organ and between clocks in different organs. This has led some to advance a federated model. On this model, there are heterarchical relations between peripheral clocks, but hierarchy vis-a-vis the SCN—which is viewed as the highest-level timekeeper that maintains its own time and is not influenced by the diverse clocks responding to different zeitgebers. In section “The SCN is different but not at the top,” I discuss recent evidence that suggests that the SCN is not above the network of other timekeepers, but part of it, engaging in 2-way interactions with peripheral timekeepers. In section “Heterarchy and local decision making,” I return to the contrast between heterarchy and hierarchy and address the concern that without a top-level agent in charge, heterarchy will lead to disorder. I conclude that mammalian circadian biology might embrace a heterarchical perspective.

## Identifying the SCN as *The Clock*

In 1960, under the auspices of the Cold Spring Harbor Symposium on Quantitative Biology, the circadian community held its first international conference. By that time, the vast majority of researchers embraced the thesis that circadian oscillations are endogenously generated. Two types of evidence were especially compelling: that rhythms persisted even when all identifiable timing cues were removed (a condition known as free-running) and that the period of these oscillations varied somewhat from 24 h (hence, the rhythms were named *circa*dian), which they presumably would not do if they were responses to external stimuli. Accordingly, many researchers directed their attention to determining what generated the rhythms—to what the title of the symposium referred to as *Biological Clocks*.

A classical approach to localizing control of a physiological or behavioral process in the brain is to show that the process is impaired when a brain region is lesioned. Adopting such an approach, Richter (1965) reported that when he lesioned the hypothalamus as a whole, circadian rhythms were destroyed. He concluded that circadian rhythms were generated in “a small area in the hypothalamus” (p. 21). Since it was known that circadian rhythms are entrainable by light, Moore traced neural pathways in rodents from the retina to the hypothalamus, identifying the retinohypothalamic pathway and showing that it terminated in the SCN (Moore and Lenn, 1972; Moore, 1973). This evidence, when coupled with evidence that lesions specific to the SCN rendered mammals arrhythmic (Moore and Eichler, 1972; Stephan and Zucker, 1972), made a compelling argument that the SCN was the locus of the circadian clock. Further evidence was provided by an SCN transplant study by Ralph et al. (1990). Taking advantage of the discovery of mutations that resulted in significantly shortened circadian periods in hamsters, these researchers transplanted the SCN from a hamster with altered rhythms into the ventricle adjacent to the SCN in a hamster whose rhythms had been normal before its SCN was removed. They found that the recipient hamster exhibited the behavioral rhythms of the mutant donor hamster. The researchers also performed the reverse experiment, transplanting the SCN of a hamster with normal circadian rhythms into one with altered rhythms before its SCN was lesioned, and showed that the hamster exhibited the normal rhythms of the donor.

After it was identified as the locus of circadian rhythms, researchers devised ways to record from SCN neurons, either by first isolating the SCN within the organism and recording from neurons near an inserted electrode (Inouye and Kawamura, 1979) or by cutting slices from the SCN and recording from neurons using microelectrodes (Green and Gillette, 1982). These studies showed increased spontaneous neural activity during daytime hours (approximately 8-9 Hz) and reduced activity during night (3-4 Hz). As the SCN was intact in these studies, researchers could not determine how it generated these rhythms—whether they were generated within individual neurons or by a circuit of neurons. By dissociating and culturing SCN cells on a multielectrode array, Welsh et al. (1995) demonstrated that individual cells remained rhythmic over a period of weeks. However, they were no longer synchronized as individual neurons varied significantly in their period (ranging from 21.25 to 26.25 h). Although synapses developed while the neurons were maintained on the array, they were not sufficient to produce synchronization. The researchers speculated that a diffusible factor may be responsible for the synchronization observed in normal slice preparation in which the neurons were not dissociated.

Subsequent research on the SCN branched in different directions. One focus was on how the individual neurons could synchronize their oscillations. Researchers differentiated populations, two of which have been primary targets of research: a population of dorsal or shell neurons that express the peptide arginine vasopressin (AVP) and a population of ventral or core neurons that express vasoactive intestinal polypeptide (VIP). Both exhibit oscillations with the same period, but differ in phase: those in the shell reach their peak firing rate earlier in the day than those in the core (Aton and Herzog, 2005).^3^ Although neurons in the core lag behind those in the shell, they are the ones that receive inputs from intrinsically photoreceptive retinal ganglion cells that were discovered to employ a distinctive opsin, melanopsin, to register light (Hattar et al., 2002; Panda et al., 2002b). Altering the phase of these neurons in response to the timing of light onset and light offset serves to entrain oscillations in the SCN to the light-dark cycle in the local environment (Hastings et al., 2018). Welsh et al. (2010) demonstrated a wave of activity traveling across the SCN, beginning with dorsomedial neurons and terminating with ventrolateral neurons.

A second focus was on how the SCN could generate daily oscillations in physiological processes and behavior. This research drew on the developing understanding of how oscillations are generated within individual cells through a feedback process involving the synthesis of proteins that, after a time delay, inhibit the transcription of the genes that code for them: the dimer of Bmal1 and Clock binds to a region (known as an E-box) on the *Per* and *Cry* genes to promote the transcription of *Per* and *Cry*, whose protein products then form a dimer and inhibit Bmal1 and Clock from binding to the E-box. This suggested that other genes, referred to as clock-controlled genes, might also contain E-boxes and be regulated in the same manner. To identify these, Panda et al. (2002a) measured the expression of more than 7000 genes in mice every 4 h and identified 650 genes that were expressed with a circadian period in either the SCN or the liver.^4^ (With respect to those whose expression was not identified as rhythmic, one cannot tell whether they were not rhythmic or insufficiently rhythmic to be detected.) Of the 650 genes whose expression they detected to be rhythmic, only 28 cycled in both organs (many of which encode core components of the clock mechanism). Panda et al. found that generally genes that were cyclically expressed in one tissue were either not expressed or only weakly expressed in the other. Through further analysis, the investigators showed that many of the genes that were detected as cycling in each tissue figured in rate-limiting steps of key biochemical pathways involved in the activities of the tissue—processing of neuropeptides and synthesis of neurotransmitters in the SCN and of nutrient transport and intermediate metabolism in the liver. (For a subsequent, large-scale atlas of clock-controlled genes, see Zhang et al., 2014.) More recently, researchers have determined that the clock also exercises control through post-translational modification of proteins (Mauvoisin, 2019; Crosby and Partch, 2020).

The discovery that gene expression in the liver and, as Panda et al. suggested, in other tissues is regulated in a circadian fashion led researches to pose the question of what mediated between the SCN and these different tissues. Silver et al. (1996) provided some clues in a follow-up to the earlier transplant study by Ralph et al. (1990). They enclosed the donor SCN in a semipermeable membrane and inserted into the ventricle, such that it was unable to form neural connections. They found that locomotor and rest-activity rhythms were restored. They attributed this to a diffusible output from the SCN. Guo et al. (2005) provided further evidence that the SCN communicated with some peripheral tissues via hormones. Since hormones travel in the blood, they connected the circulatory system of animals with and without an SCN and found this resulted in circadian behavioral rhythms in liver and kidney in the animal without an SCN.

**Figure 1.**
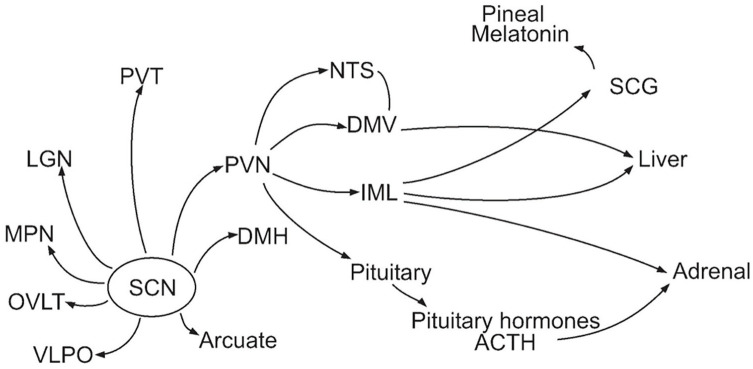
Neural and endocrine projections from the SCN (Panda and Hogenesch, 2004). Abbreviations: SCG = superior cervical ganglion; IML = intermediolateral nucleus.

However, only rhythms of some physiological processes were restored in these experiments, suggesting that others depended on neuronal projections from the SCN. Using strategies of neural tracing similar to those employed by Moore to reveal projections from the retina to the SCN, researchers identified a host of neural projections from the SCN. As reviewed by Panda and Hogenesch (2004) and shown in Figure 1, neurons from the SCN project to numerous adjacent nuclei of the hypothalamus—the paraventricular nucleus (PVN), the dorsal medial hypothalamus (DMH), ventrolateral preoptic area (VLPO),^5^ and the arcuate nucleus (ARC)—as well as the lateral geniculate nucleus (LGN) and paraventricular nucleus of the thalamus (PVT) and the organum vasculosum lamina terminalis surrounding the third ventricle (OVLT). Of these, the PVN is a particularly important integrative locus for both autonomic neural projections (e.g. to adrenal, liver, kidney, pancreas, adipose tissue, and heart) and neuroendocrine release. Corticotropin-releasing factor-producing neurons in PVN govern adrenocorticotropic hormone release from the pituitary, which then regulates glucocorticoid release from the adrenal glands (Buijs et al., 2003).^6^ These pathways provide a plausible mechanism for the distribution of timing information from the SCN.

The research discussed in this section exemplifies research on biological mechanisms more generally—a function (circadian rhythmicity) is identified, localized to a particular tissue (the SCN), and researchers developed accounts of mechanisms within that tissue, inputs from other tissues (retinal ganglion cells) and outputs from it to other parts of the organism (brain areas or organs of the body). As described by Bechtel and Richardson (1993/2010), such research is based on a heuristic assumption of localized control, an assumption that is fallible. In the following sections, I turn to research that has pushed beyond that initial assumption and offered a far more complex, less localized, account of the phenomenon of circadian rhythmicity.

## Clockworks but not Autonomous Clocks in Every Tissue

The accounts described so far did not identify how signals from SCN produce rhythmic physiological processes in peripheral tissues. An important insight resulted from the research that identified the genes and proteins that generate oscillations in individual SCN neurons. Researchers determined that the same genes are expressed in peripheral tissues such as the liver, kidney, and lungs. That the genes are expressed, however, does not show that they produce circadian oscillations. Working with rat fibroblasts that had been maintained for 25 years with no contact with an SCN and with rat hepatoma cells, Balsalobre et al. (1998) found that applying a serum shock generated 24-h oscillations (measured in terms of expression of *Dbp*, a transcription factor, as well as for other clock-related genes). However, the rhythms dampened after the first cycle and disappeared over about 3 days. Based on these results, they concluded “peripheral tissues contain a clock capable of measuring time with impressive precision” (p. 934). However, the fact that these rhythms rapidly dampened suggested that peripheral tissues such as the liver only oscillated when they received the equivalent of a serum shock from the SCN. In a subsequent study, Zylka et al. (1998) offered further evidence suggestive of circadian oscillations in peripheral tissues in mice when they demonstrated that expression of all 3 mammalian *Pers* exhibited circadian oscillations in skeletal muscles and testes.

One question arising from these studies was what, in living organisms, played the role of serum shock in Balsalobre et al.’s study. In a subsequent paper, Balsalobre et al. (2000) proposed that glucocorticoids might play that role. Specifically, they proposed that neural outputs from the SCN to tissues such as the PVN initiated a process leading to the release of glucocorticoids from the adrenal glands that could then act on glucocorticoid receptors. There are such receptors on most peripheral cell types but none on SCN neurons, making the signal transmission one-directional. In support of this hypothesis, the researchers showed that injections of the glucocorticoid dexamethasone could cause phase shifts in circadian rhythms in the livers of mice, an effect that was absent in the livers of mice in which the glucocorticoid receptor was inactivated. More recently, Soták et al. (2016) found that removal of the adrenal gland did not affect the phase of the expression of peripheral clock genes in the various peripheral tissues they studied, but did significantly alter the amplitude of oscillations of different clock genes in different tissues. In liver, it downregulated *Per1* and *Cry1* and upregulated *Rev-Erbα* and *Rorα* (for a review, see Zhang et al., 2020). However, since clock genes in the livers of mice with inactivated glucocorticoid receptors still followed the lead of the SCN, Balsalobre et al. concluded there must be other signals determining the phase of peripheral clocks. As liver cells have melatonin receptors, researchers have investigated its potential in signaling to the liver. Although knockout of melatonin receptors only produced slight effects on the phase of clock genes in the liver, Mühlbauer et al. (2009) found that knockout of the MT1 receptor resulted in a large decrease in the amplitude of *Per1* expression rhythms while knockout of the MT2 receptors resulted in increase in the amplitude of Rev-Erbα expression rhythms. Part of the challenge in determining what timing signal is transmitted from the SCN to peripheral tissues is that multiple signals may contribute in different ways.

Balsalobre et al.’s identification of oscillations involving clock genes in peripheral tissues filled an important gap in the account of how the SCN regulates physiology and behavior—on their account, signals from the SCN elicit these oscillations, which then generate the rhythmic expression of specific other proteins in different peripheral tissues. On Balsalobre’s account, there is an important difference between these oscillations in peripheral tissues and those in the SCN—those in peripheral tissues quickly dampen without inputs from the SCN. Peripheral clocks are directly under the control of the SCN.

## Endogenous Clocks in Peripheral Tissues that Require the SCN for Entrainment

The studies indicating that the clockworks in peripheral tissues did not generate rhythms when not driven by the SCN measured gene expression in populations of cells, either in cultured or in peripheral tissues. An alternative strategy is to use a reporter of circadian clock gene expression that enables recording from individual cells. Using a bioluminescent recorder, Yamazaki et al. (2000) found rhythms in peripheral tissues in culture, but in their studies too rhythms damped out after a few days. Using an improved circadian reporter in which the clock protein PER2 is fused to firefly luciferase (PER2::LUC), Yoo et al. (2004) showed more persistent circadian oscillations of peripheral tissues in culture. A few months later, Welsh et al. (2004) and Nagoshi et al. (2004) used single-cell imaging of bioluminescent or fluorescent circadian reporters to show that individual fibroblast cells continued to oscillate indefinitely in culture. As with dissociated SCN cells, fibroblast cells lost synchrony with each other after a few days in culture when undisturbed, so that their phases were randomly distributed across the day. This loss of synchrony explains the apparent loss of rhythmicity in the population. As Welsh et al. (2004, p. 2294) note, the upshot is that “the SCN in vivo may serve to synchronize peripheral clock cells to one another and to the light/dark cycle, although it may not be required to sustain their oscillations.”^7^ On this account, the SCN is not driving the peripheral oscillations.

At this point, Davidson et al. (2004) proposed a change in metaphors to capture the changing understanding of the relation of the SCN to peripheral clocks. The previous understanding he termed the “circus ringmaster” view according to which the ringmaster (SCN) drives animals to behavior. Without the ringmaster, “Horses will not circle the ring—and tigers will refuse to jump through flaming hoops” (p. 110). Yoo et al. and Welsh et al. had discredited this metaphor by showing continued oscillation in peripheral tissues. According to the new metaphor Davidson et al. proposed, peripheral clocks are like orchestra players who can play their instruments, but with “each musician keeping his own time until they drift gradually out of synchrony and the music fails” (Davidson et al., 2004). The role of the orchestra conductor is to provide a signal that enables the players to synchronize. Davidson et al. proposed the “SCN may indeed be the conductor of an orchestra composed of dozens, if not thousands of potentially independent oscillators” (p. 111).

Discovering oscillation of circadian clock genes in peripheral tissues, however, does not establish that these peripheral clocks are playing any role in circadian regulation of the organism. Their oscillations could be entirely epiphenomenal. One way to establish that a component plays a role in a process is to lesion it and demonstrate that the process is impaired. To incapacitate peripheral clocks, researchers interfered with their molecular operation—for example, by knocking out a core gene such as *Bmal1* (the one clock gene whose incapacitation alone eliminates circadian rhythmicity). An organism-wide knock out of *Bmal1* would affect the SCN and all peripheral clocks. Thus, to show the role of the circadian clock in the liver, Lamia et al. (2008) generated a mouse line lacking *Bmal1* just in the liver. They found that these mice failed to increase the concentrations of the enzyme GLUT2, which is responsible for glucose export from the liver, during their normal fasting (inactive) period. Not surprisingly, the mice are hypoglycemic during that period. The authors conclude

our findings strongly suggest that, in addition to well described acute hepatic responses to circulating glucose levels, the liver circadian clock drives a daily rhythm of hepatic glucose export timed so as to counterbalance the brain-driven fasting—feeding cycle, thereby buffering blood glucose concentrations over the course of the daily behavioral cycle. (p. 5)

Similarly, researchers have demonstrated that circadian clocks in other peripheral tissues play important roles in the physiology of those tissues. Like the liver, the pancreas plays an important role in blood glucose regulation. By knocking out *Bmal1* specifically in the pancreas, Marcheva et al. (2010) demonstrated reduced insulin secretion, resulting in increased blood glucose levels throughout the day and impaired glucose tolerance (see also Sadacca et al., 2011; Perelis et al., 2015). Eliminating Bmal1 just in muscle, Dyar et al. (2014) showed impaired glucose metabolism, including impaired insulin-stimulated glucose uptake and reduced glucose oxidation. Doing the same in adipose tissue, which normally stores excess energy in triglycerides, Paschos et al. (2012) demonstrated reduced polyunsaturated fatty acids in adipocyte triglycerides. Employing a mutation of a different circadian gene, *Clock*, Ko et al. (2011) demonstrated effects of the cardiac clock on the ability of mice to increase their running wheel activity over time and traced the effect to altered phosphorylation states of several kinases as well as to loss of a voltage-gated calcium channel. Welz et al. (2019) showed that clocks in the skin maintain basic functions such as epidermal turnover when the clock is restored just in that tissue. Taken together, these studies reveal that peripheral clocks play important roles in regulating the activities of their respective tissues.

The results of these various lines of research all seem to support the orchestra conductor model: cells in peripheral tissues are capable of maintaining oscillations that regulate physiological activities in those tissues but, without the SCN, cannot maintain synchrony. When synchronized by the SCN, the cells in different tissues operate as clocks, generating circadian rhythmicity in the activities of the tissues.

## Alternative Entrainment for Endogenous Clocks

The orchestra conductor model maintains the hierarchical understanding of the relation between the SCN and peripheral timekeepers—like orchestra players who each know how to play their own instrument, the peripheral clocks are able to keep time and regulate activities in their respective tissues by regulating clock-controlled genes. If they are really like orchestra players, however, one might imagine that peripheral clocks could exhibit more independence. Orchestra players are typically not just automata, each simply playing on command. Musicians bring their own mode of playing to the orchestra and, moreover, can adapt their playing to the conditions in the performance space. The corresponding feature of peripheral clocks would be to respond to different zeitgebers, not just the SCN.

Light is not the only zeitgeber. There are other conditions in the environment that correlate with the day-night cycle and to which organisms are responsive. One is ambient temperature. An important feature of the circadian clock is that it is temperature compensated—unlike most mechanisms relying on biochemical reactions that proceed faster in warmer temperatures, the circadian clock maintains the same oscillatory period regardless of temperature. But it can receive signals from other temperature sensors, and many animals have sensors for temperature either at the skin or within their body. The rhythm of temperature can serve as a zeitgeber, even for fibroblasts (Brown et al., 2002; Schibler, 2024). The conditions that afford activities such as locomotion and eating for a given species also tend to be limited to particular periods in the day-night cycle—for example, prey may be available only at particular times. These activities, for example, meal timing, can then also serve as zeitgebers. While non-light zeitgebers tend to correlate with the presence or absence of light, they can deviate. When they do so, it may be beneficial to the animal to attend to them. If an animal that is usually active at night finds itself in an environment where food is available only during the day, then that is when it will eat. But if its physiology is being regulated by the light-dark cycle, it will not have synthesized the enzymes needed to process food. Of particular concern, it may lack the enzymes to convert excess glucose to glycogen and experience hyperglycemia (it may also face hypoglycemia during its normal active period when it is expecting a bolus of glucose but does not receive it). As the liver is particularly important for regulating glucose, in this scenario, it would be advantageous to the animal (at least in the short-term) if the circadian oscillators in the liver used eating as a zeitgeber and oscillated in accord with it, deviating from the signal from the SCN.^8^ This would be like an orchestra player who altered his or her playing in response to a signal other than that from the conductor.

Circadian researchers have long recognized that availability of food serves as an independent zeitgeber for endogenous rhythmicity in many animals. While researchers were still establishing that circadian oscillations were endogenous, Richter (1922) fed rats only during periods outside their normal active period (during the night) and monitored their running wheel activity. Although rats normally run on wheels only during their active period, when they were fed regularly only during the day, they also ran in the period prior to that feeding time. Much later, Stephan (1992, 2002; Stephan et al., 1979; Stephan and Zucker, 1972) elaborated on these studies, showing that not only locomotion but also core body temperature and corticosterone secretion shifted to track food availability. Since by this time researchers had identified that SCN as the locus of a light-entrained oscillator, Stephan and colleagues hypothesized a comparable food-entrainable oscillator (FEO).

Despite intense testing of different hypothesized sites, the FEO has not been located. But once clockworks were identified in peripheral tissues, some researchers took advantage of alternative feeding times to show that peripheral clocks can be entrained by these zeitgebers. Damiola et al. (2000) demonstrated that, regardless of whether mice were maintained on a light-dark cycle or in constant darkness, when, over the course of 1 week, they were fed during what would have been their inactive phase, expression of clock genes and clock controlled genes first in the liver but subsequently in the kidney, heart, and pancreas, decoupled from those of the SCN (whose rhythms were unaffected) and followed the feeding regime.^9^ Using a luciferase knock-in to register *Per1* expression, Stokkan, Yamazaki, Tei, Sakaki, and Menaker (2001) showed a 10-h advance in gene expression in the liver within 2 days and a full 12-h adjustment after 7 days.^10^ They showed that the lungs responded more slowly, shifting by 6 h after 7 days. Again, this had no effect on the SCN.^11^

Stokkan et al. interpret their results as seriously challenging the assumed hierarchical model in which the SCN drives or entrains peripheral rhythms. Their data, they assert, show “that the phase of circadian rhythmicity in the liver is independent of both the SCN and the light cycle under conditions that are no less ‘normal’ than those that usually prevail in the laboratory” (p. 492). Beyond demonstrating these effects of manipulating feeding, they propose that these altered feeding experiments indicate that the SCN normally acts on the liver not by direct neural or endocrine signaling but by regulating feeding behavior and, accordingly, the time when food is consumed. They present this as “a view that places at least one link in the causal chain [of entraining the liver clock] completely outside the animal” (p. 492.).

A key question raised by the discovery that feeding time can entrain the liver clock is how the liver acquires information about feeding time. A variety of signaling molecules are known to affect expression of core clock genes (reviewed in Tahara and Shibata, 2018). A common pretreatment for time-restricted feeding is fasting, which triggers release of numerous signaling molecules, including glucagon, not only by the liver but also by the pancreas and kidneys. Fasting acts on the liver clock by activating cAMP response element-binding protein (CREB), which in turn induces *Per1* and *Per2* expression. After 24 h of fasting, glucagon also promotes *Bmal1* synthesis. The reduced blood glucose resulting from fasting and also activates AMP-activated protein kinase (AMPK), which phosphorylates Cry1, leading to its degradation in the nucleus. Subsequent eating induces insulin production from the pancreas, which increases *Per 2* expression not only in the liver but also in the white adipose tissue. Finally, oxyntomodulin, produced in the gut, increases both *Per1* and *Per2* expression in the liver.

Researchers focusing on clocks in organs other than the liver have also identified zeitgebers that can set the timing of those clocks. For example, Yamanaka et al. (2008) demonstrated that timing of exercise provides a zeitgeber for clocks in skeletal muscles. The researchers simulated time-zone change by advancing the light period by 8 h for 3 days and then maintaining mice in constant darkness. This sufficed to advance gene expression in the SCN, but the muscle and lung clocks only fully advanced to the new period when a running wheel was available during the new dark phase. Following up on that study, Wolff and Esser (2012) showed that, without changing the LD cycle, exercise timed to the inactive period resulted in shifted circadian rhythms in 3 different skeletal muscles (the flexor digitorum brevis, the extensor digitorum longus, and the soleus). Likewise, the clocks in the lungs are altered by hypoxia (Manella et al., 2020).

Manella et al. (2021) found that while daytime feeding in mice resulted in reduced amplitude of clock gene expression in different tissues, the change in phase of gene expression differed among the tissues. When feeding in mice is restricted to daytime, clock gene expression in both the liver and white adipose tissue shifted a full 12 h. Expression of clock genes in other tissues such as kidney and heart made a less complete shift of 4-8 h. Genes in lung tissue did not shift, whereas clock genes in muscle tissue ceased to be rhythmic.^12^ The investigators further found that clock rhythms in liver during daytime feeding correlated with rhythmic expression of the whole transcriptome. In contrast, in white adipose tissue, the phase shift varied, with some proteins aligning with the feeding time and the clock, but others not shifting, or only partially shifting. They concluded that “feeding time affects clock rhythmicity in a tissue-specific manner. . . . [I]t appears that [daytime feeding] not only uncouples peripheral clocks from the central clock but also uncouples clocks of different peripheral tissues” (p. 832).

The original studies demonstrating that peripheral clocks can be entrained by feeding time used animals without a functioning SCN. But these more recent studies have shown that entrainment to feeding time in peripheral tissues occurs even in the presence of a functioning SCN as well. An important difference is that entrainment when the SCN is functional is much slower (Saini et al., 2013)—as noted above, the liver clock adjusts after 6 days when the SCN is lesioned but requires much longer when it is present. This indicates that both the SCN and feeding act on the liver clock and has led some theorists to propose that the SCN, rather than providing the primary timing signal to the liver clock, acts to stabilize its activity (preventing it from adapting too quickly to aberrant feeding times that may be temporary). Note that this represents a significant change from the hierarchical perspective—on this view, the SCN provides an input to the peripheral clock, but it does not direct its activity (which is primarily responsive to the local zeitgeber, feeding time).

The evidence that peripheral clocks respond to zeitgebers appropriate to the activity of the tissues in which they reside can be fitted to the orchestra conductor model—orchestra players are not automata and maintain their own rhythms that can be constrained by the conductor. But it provides a much richer conception of peripheral clocks than was invoked by Davidson et al. when they first proposed their model. Each peripheral clock maintains its own rhythmicity, altering its activity in response to various zeitgebers. The SCN may be especially influential, but peripheral clocks have the resources to entrain to other zeitgebers.

## Peripheral Clocks can Coordinate Themselves without the SCN

The research discussed so far has focused on individual peripheral clocks maintaining rhythmicity and being entrained by zeitgebers appropriate to the specific organ in which they reside. Recent research has advanced evidence that, both within tissues and between tissues, peripheral clocks coordinate their timekeeping. The orchestra does not seem to be populated by thousands (as suggested by Davidson et al.) of individual players who each only attend to the SCN, but by players who take cues from each other and form ensembles. As I discuss at the end of the section, this challenges Davidson orchestra conductor model and has prompted introduction of the federated model as an alternative.

A starting point for research on coordination of peripheral clocks was a study by Tahara et al. (2012) that showed that even without the SCN, half the mice from which they recorded exhibited sustained rhythms in kidney, liver, and submandibular gland. To examine this capacity in the liver in more detail, Sinturel et al. (2021) used luciferase reporters to monitor rhythms in the livers of freely moving SCN-lesioned mice in constant conditions and ad libitum feeding, and found that both Rev-erbα and Bmal1 maintained oscillations, albeit with slightly greater variability in period length than in mice with an intact SCN. The researchers then created mice with a complete complement of clock genes only in hepatocyte cells^13^ and again used a luciferase reporter linked to Rev-erbα. These animals also exhibited sustained rhythmicity, albeit less coherent, suggesting less synchronization of liver cells than in mice with the clock only deleted in the SCN. They propose: “Conceivably, in SCN-lesioned mice, signals emitted by liver cells other than hepatocytes (or nonhepatic tissues) might have participated in the synchronization of hepatocyte clocks” (p. 332).

In their study, Sinturel et al. only found ongoing circadian rhythmicity in the liver. When they measured bioluminescence organism-wide in SCN-lesioned mice kept in constant conditions, the rhythms elsewhere were severely dampened. The researchers inferred that while cells in the liver are interconnected, those in different organs are not and are dependent on signals from the SCN to remain synchronized. Other research suggests coupling between cells in other peripheral clocks. Using in vitro models of human peripheral clocks (primarily human osteosarcoma cells), Finger et al. (2021) demonstrated coupling between clocks by showing phase adjustments when cells with different circadian phases were mixed. They also demonstrated period adjustments when cells with different intrinsic periods were cocultured in 3-dimensional spheres. By establishing that the synchronization required intact Golgi apparatus and endoplasmic reticulum, they provided evidence that it depended on paracrine signals. Finding transforming growth factor-β (TGF-β) to be present in all of the fractions they investigated, they focused on it and provided additional supporting evidence that it was the synchronizing signal. While chemicals constitute one potential mode of communication between cells in a tissue, physical structures such as the extracellular matrix (ECM) provide another. Koronowski and Sassone-Corsi (2021) report that when hepatocytes are cultured on ECM structures, they maintain more robust rhythms—while they still lose amplitude, they maintain their phase relation.

Yet additional research points to a variety of ways in which clocks in different tissues interact. One clue to such interaction is that not all activities in a given tissue respond to clocks in that tissue. Above I described research that knocked out the essential clock gene *Bmal1* in just one tissue to determine what functions were impaired. Koronowski et al. (2019) employed the reverse strategy—knocking it out globally and then restoring it just in the liver. They found that when the liver received a zeitgeber by time-restricted feeding, rhythmicity was restored to only some liver activities, notably those involved in regulating blood glucose levels such as glycogen synthesis and NAD^+^ salvage production. But the liver performs many other functions, and rhythmic performance of these activities was not restored.^14^ In addition, Greco et al. (2021) found that liver activities tied to redox and lipid metabolism were not regulated by the liver clock but by the clock in skeletal muscle.

Other research has identified how clocks in the liver are regulated by other tissues. For example, Petrenko et al. (2017) demonstrated that circadian clocks in α- and β-cells in the pancreas have opposite phases. These regulate glucagon and insulin, respectively, both of which act on clocks in the liver. Glucagon acts on adenylate cyclase, promoting synthesis of cAMP, which in turn activates CREB. CREB in turn activates *Per1* and *Per2* expression. Insulin acts in the liver to alter Bmal1 accumulation in the nucleus of liver cells (Dang et al., 2016).^15^ Clocks in the pancreas are thus able to modulate activity of the liver clock. As another example of clocks in other tissues acting on the liver clock, Landgraf et al. (2017) showed that signaling peptides released under the influence of clocks in the gastrointestinal tract (ghrelin in the absence of food and oxyntomodulin after feeding) also act on the liver clock.

Other research by Manella et al. (2021) demonstrated the converse: that the clock in the liver affects activities in other tissues. When they knocked out *Bmal1* just in the liver, the transcriptome in white adipose tissue and the lungs no longer adjusted to daytime feeding. Since the clocks in those tissues were not affected, transcriptional changes in those tissues had to be due to the changes in the liver clock. The researchers propose that this effect is mediated by metabolites produced by temporally altered glucose metabolism in the liver as well as other metabolites, peptides, and hormones that are under circadian control in the liver.

A recent study by Smith et al. (2023a) revealed additional complexity in the interactive effects of clocks in different tissues. In mice lacking circadian oscillations organism-wide, the researchers reconstituted the clock in either just the liver, just skeletal muscle, or both. When the clock was restored in each tissue separately, only some of the typically oscillating transcripts oscillated. In many cases, those that oscillated did so with altered phase and amplitude. Smith et al. did find a degree of cross-tissue regulation, with some muscle genes becoming rhythmic only when the clock in both liver and muscle were rescued. They did not find this surprising given the extensive interactivity of muscle and liver in executing critical metabolic activities. But they also demonstrated that rescuing just the clock in liver and muscle was not sufficient to generate normal transcriptomic rhythms. Since one of the rhythmic behaviors that is lost without a functioning SCN clock is feeding, the researchers added a condition in which they restricted feeding to the night, with or without rescuing the liver and muscle clock. While overall restricted feeding sufficed to rescue many rhythms, rescue of some rhythms involved in glucose homeostasis required both restricted feeding and rescue of the clock in both muscle and liver. In particular, the researchers identified effects of clock rescue in liver and muscle on rapid metabolism of glucose by muscles and clearance of the derived metabolites of glucose by the liver. They conclude that “a daily feeding-fasting rhythm is a key input to liver and muscle clocks that enables synergy between the two tissues for control of glucose metabolism at the systemic level” (p. 12).^16^

**Figure 2.**
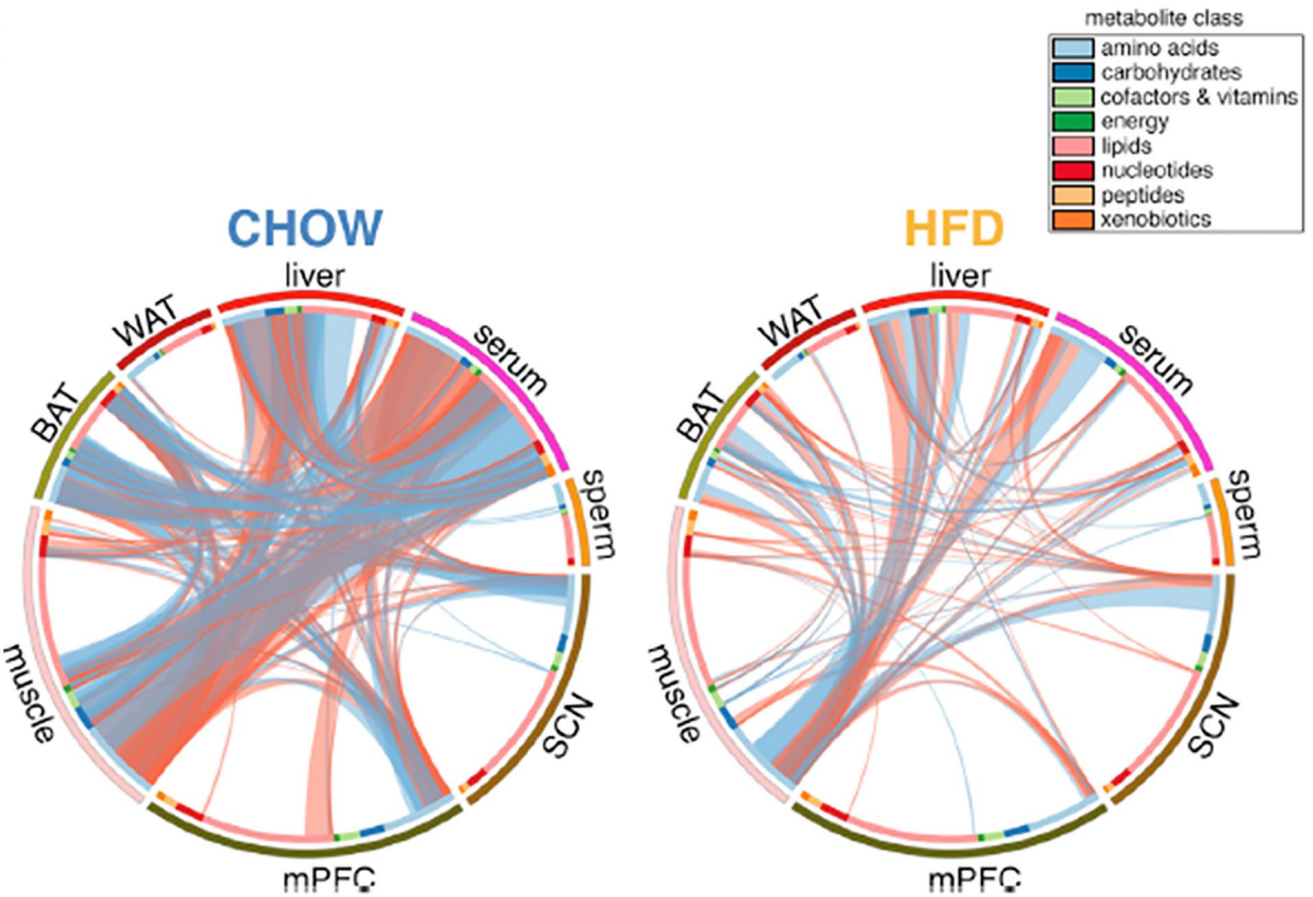
Correlations of metabolites across 8 different tissues in mice fed either regular chow (left) or a high-fat diet (right). From Dyar et al. (2018).

To provide a more comprehensive picture of how metabolites produced in specific tissues could coordinate peripheral clocks in other tissues, Dyar et al. (2018) developed global metabolic profiles for 8 tissues (liver, brown and white adipose tissue, muscle, sperm, blood, prefrontal cortex, and the SCN) at 4-h intervals and correlated the results across tissues. They interpreted the correlations as indicating metabolic coupling. By feeding some mice regular chow and others a high-fat diet, they were able to show how the correlations changed depending on the diet. They found that with normal chow, there are rich correlations between muscle, brown adipose tissue, liver, and blood that are radically reduced with a high-fat diet (Figure 2). The results suggest that metabolite concentrations can function to coordinate clocks in different peripheral tissues.

In addition to hormones and metabolites transported through the bloodstream and other fluids of the body, recent evidence demonstrates that messages from one tissue to another can be conveyed by the contents of extracellular vesicles, especially exosomes (Yeung et al., 2022). These vesicles contain a variety of molecules including membrane and cytosolic proteins, mRNAs, and non-coding RNAs (Tao and Guo, 2018). The contents of the vesicles can act on membrane receptors of target cells, but extracellular vesicles can also be taken up into other cells, allowing their contents to act on intracellular targets. These vesicles have been found to contain many molecules that are known to have effects on circadian clocks. For example, SIRT1, a product of glucose metabolism, figures in the regulation of expression of *Per* genes. Likewise, phosphorylated GSK-3 acts on CRYs and the Bmal1::Clock dimer, while AMPK acts on CRYs.

Peripheral clocks can not only act directly on other tissues and clocks in those tissues through hormones and metabolites that they release, but also through neuronal signaling systems that have organism-wide effects. For example, they can signal through one of the main signaling systems employed by the SCN—the PVN. As discussed above, neurons in the PVN regulate release of glucocorticoids from the adrenal gland. The SCN, however, is not the only hypothalamic source of signals to the PVN. Both the dorsomedial hypothalamus and the medial preoptic area send inhibitory projections to the PVN while the arcuate nucleus sends excitatory projections. Signals from peripheral clocks reach these hypothalamic nuclei (e.g. insulin, ghrelin, and leptin all act on the arcuate nucleus) and can affect glucocorticoid signaling.

**Figure 3.**
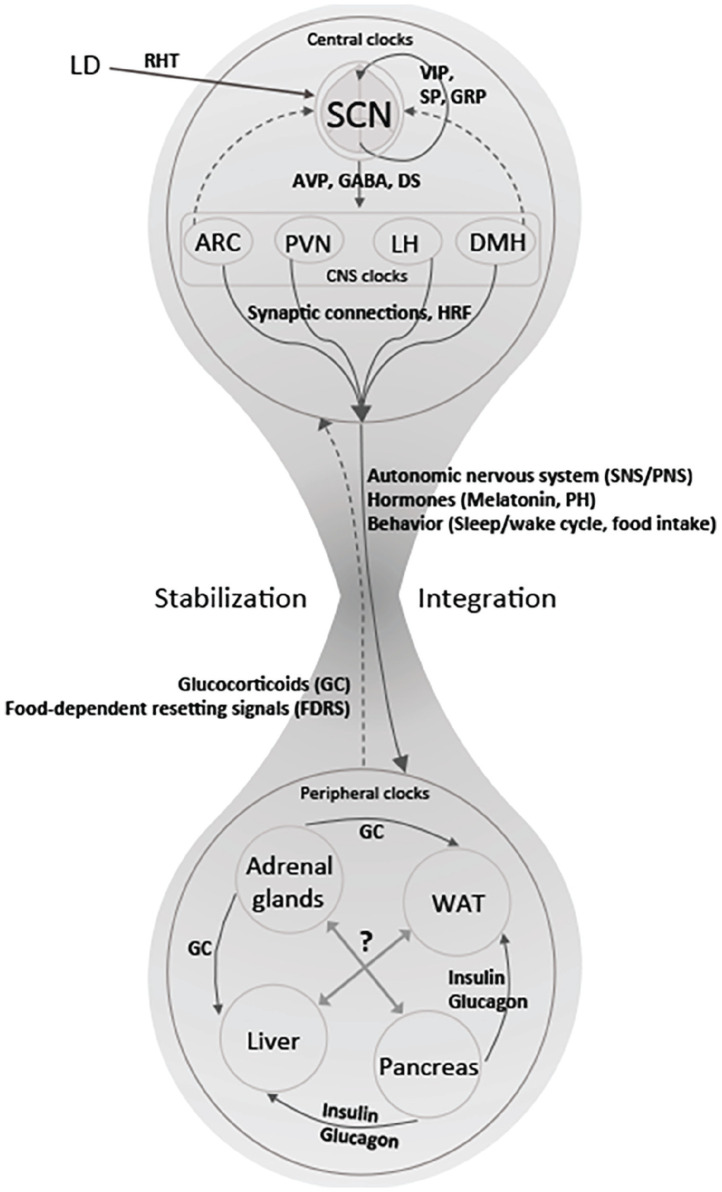
The federated model: Peripheral clocks maintain their own rhythms and coordinate their activities through various peptides. The SCN, operating through clocks in other hypothalamic tissues, sends signals to peripheral oscillators. From Astiz et al. (2019).

In light of findings such as these demonstrating communication among peripheral clocks, Oster and his collaborators have advanced what they term a *federated model* (Figure 3). They don’t explain the motivation for the name, but it is clear that they view it as characterizing local clocks as controlling specific phenomena while also sharing information:

A “federated” network, however, allows for each clock and, therefore, each physiological process to be synchronized to those zeitgeber signals that are most relevant for a particular process, resulting in a tailored response. For example, the liver clock should be synchronized to rhythms in food intake, but it should also respond to changes in energy demands or variations in oxygen supply. A “federated” organization allows for better adaptation to changing environments. (Husse et al., 2015)

A major feature of the federated model is to downplay the role of the SCN. According to de Assis and Oster (2021), the federated model

suggests that the SCN is only required to sustain the rhythms of the organism in the absence of zeitgebers or under partially conflicting zeitgeber conditions, which resembles a hierarchical structure. This situation changes when reliable zeitgebers are present, when peripheral organs are able to sustain their rhythms in a SCN-independent fashion, through a tissue-specific combination of zeitgebers, which is expected to allow for more flexible entrainment under complex zeitgeber conditions than a strict hierarchical structure.

The federated model construes the peripheral clocks as fully functioning clocks drawing upon different zeitgebers and coordinating their timekeeping. It retains a hierarchical component in that it construes the SCN as the preeminent timekeeper: although it allows significant autonomy to peripheral clocks, it asserts itself when zeitgebers to peripheral clocks fail or conflict. It remains the final arbiter.

## The SCN is Different but not at the Top

On the federated model, the SCN is viewed as largely independent of the interactions of the peripheral clocks, and thus, as in Figure 3, is envisaged as at the top of the hierarchy, ready to step in when peripheral clocks fail or might be in error. Possible ways in which the peripheral clocks can influence the hypothalamus, and in which other clocks in the hypothalamus can influence the SCN, are indicated only with dashed lines. In part, the assignment of the primary role in circadian timekeeping to the SCN is based on the assumption that the SCN is the most reliable clock in that its timing only reflects the environmental light-dark cycle and is not changed by other zeitgebers. For instance, with time-restricted feeding other clocks altered their phase, but the SCN remained true to the light-dark cycle. While the SCN could not prevent peripheral clocks from entraining to other zeitgebers, it did limit the response, and so was viewed as stabilizing the peripheral clocks (Acosta-Galvan et al., 2011).

In this section, I will review recent evidence that suggests that the SCN is not so oblivious to other sources of timing information. It has access to information other than light, especially information about the metabolic state of the body. While it is still unclear how the SCN responds to this information, it is highly plausible that it does so and that its role is more like that of other clocks—primarily responsive to a specific zeitgeber but influenced by others. As I discuss at the end of the section, rather than being in a privileged position at the top of the hierarchy, it may be part of a heterarchical network of timekeepers.

Given the important role that meal timing has played in revealing the role of peripheral clocks, much of the information about how the SCN is influenced by other zeitgebers has focused on signals related to eating. Contrary to the claims that restricting feeding to the inactive period does not affect the SCN, Dattolo et al. (2016) demonstrated that SCN neural activity, which is normally high during the inactive period, is reduced before and during feeding when it is restricted to the inactive period. Furthermore, fasting has been shown to reduce electrical activity in the SCN, which Saderi et al. (2013) trace to neuropeptide Y (NPY) projections from the intergeniculate leaflet (IGL), that is in turn responsive to serotonin signals.^17^ Buijs et al. (2017) also found that the SCN is itself responsive to glucose—oral glucose administration reduces SCN activity.

Ghrelin and leptin are 2 peptides that play an important role in signaling the nutritional status of animals. The main sources of ghrelin in mammals are specialized ghrelin cells in the lining of the stomach^18^ and early parts of the small intestine (they are also found in smaller quantities in tissues such as the liver, pancreas, and lung). These cells release ghrelin by default, but cease to do so promptly on eating (or even anticipation of eating). White adipose tissues, fat cells located underneath the skin and surrounding internal organs, as well as the lining of the stomach are the major source of leptin, which is secreted in proportion to fat mass. I focus on 2 ways these peptides signal to the SCN. First, the SCN has receptors for both peptides (for leptin, see Bookout et al., 2013; for ghrelin see Zigman et al., 2006), allowing for direct signaling. Although there is no direct evidence as to how the SCN responds to these signals, elsewhere ghrelin has been shown to act on growth hormone secretagogue receptors (GHSRs) to delay Per expression. This may mediate the phase delay Zhou et al. (2014) demonstrated with administration of a bolus of ghrelin to mice at the beginning of their subjective night. Kulkarni et al. (2023) reported that “ghrelin has a maximal impact on SCN neuronal activity during the resting phase when rodents are housed under LD conditions and during the active phase when rodents are housed under DD conditions.” Since ghrelin release from the stomach is regulated by the stomach clock, which is responsive to feeding time, Yannielli et al. (2007) suggest this may facilitate communication between the stomach clock and the SCN. In contrast, leptin has also been shown to act on SCN slices, producing a phase advance in a dose-dependent manner (Prosser and Bergeron, 2003).

Second, ghrelin and leptin are two of the major molecules signaling to the arcuate nucleus (ARC) of the hypothalamus, a major center for processing nutritional information (Jais and Brüning, 2022) and in which individual neurons can maintain circadian oscillations and synchronize their activities with each other (Guilding et al., 2009). Agouti-related peptide (AgRP) and neuropeptide Y (NPY) neurons have GHSRs and, in response to stimulation by ghrelin, activate orexin-releasing neurons in the lateral hypothalamus that promote feeding activity and inhibit feeding suppressing neurons in the PVN. These neurons also have receptors for both leptin (and insulin), which act to inhibit them.^19^ In contrast, alpha melanocyte-stimulating hormone (α-MSH) neurons, a subset of proopiomelanocortin (POMC) and cocaine and amphetamine regulated transcript (CART) coexpressing neurons, are activated by leptin and insulin. They in turn activate PVN neurons to terminate food intake while inhibiting orexin neurons in the lateral hypothalamic area (LHA). In addition, GABA projections from the AgRP/NPY neurons act to inhibit α-MSH neurons. As a result of these interconnections, the ARC is the locus of a competition between populations of neurons responsive to opposing signals about the animal’s nutritional state, with the resulting output to other hypothalamic area determining feeding behavior (Garfield et al., 2015).

**Figure 4.**
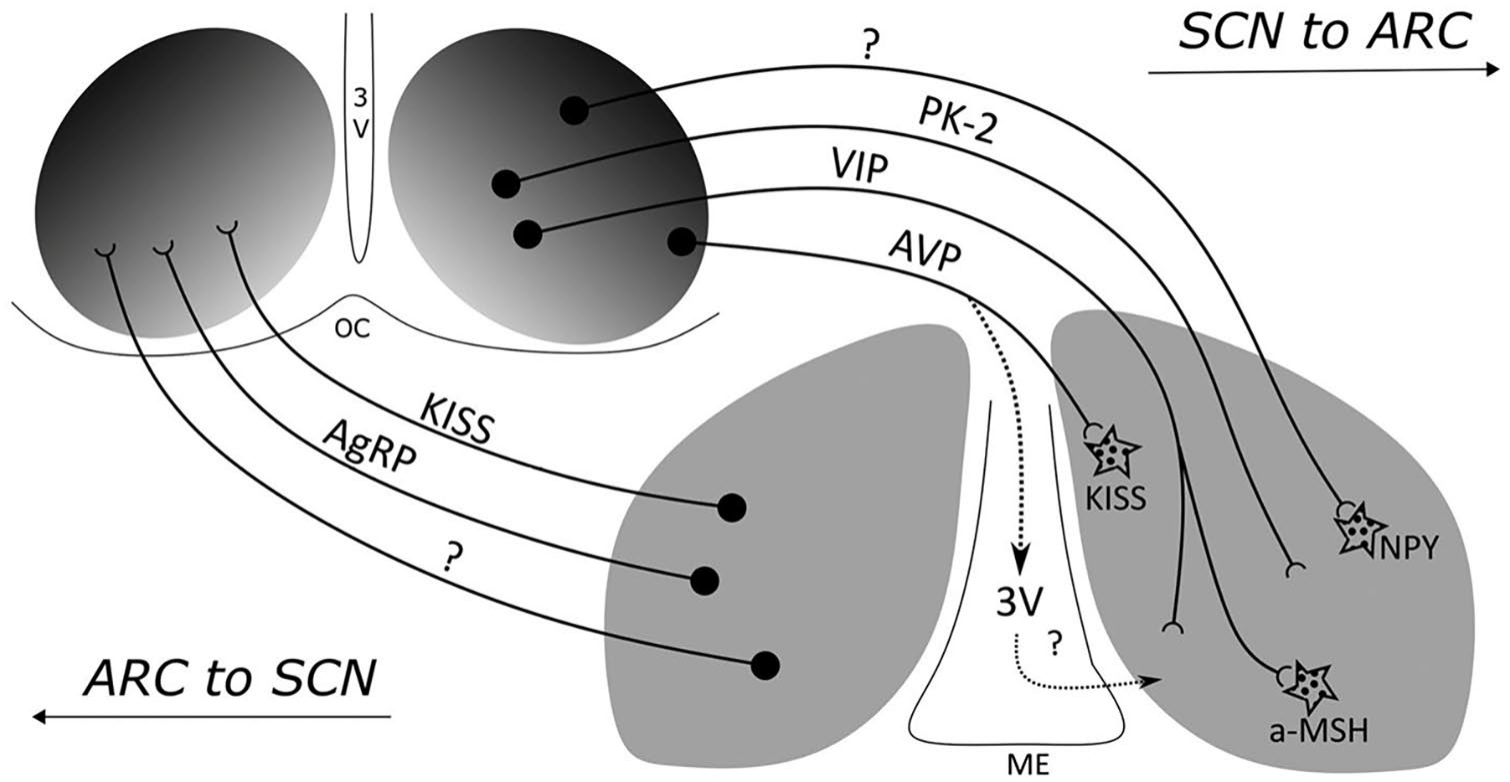
Two-way communication between the SCN and the ARC. Different transmitters figure in the communication in the 2 directions. From Méndez-Hernández et al. (2020).

Although the activity of the ARC is often described without reference to circadian rhythms, it exhibits circadian activity. Unlike the SCN, its overall activity peaks at night. These oscillations result from several known and hypothesized projections from the SCN to the ARC (Figure 4). One well-documented pathway is from VIP neurons in the SCN to the α-MSH neurons, enhancing their responsiveness to leptin at the end of the dark phase in mice even when they are fasted (Guzmán-Ruiz et al., 2014). When these neurons are lesioned, the amplitude of the oscillation in food intake is dampened and totally eliminated in constant darkness. Although the source of projections to NPY neurons is not known, they exhibit rhythmicity in both the ARC and the PVN, peaking at the onset of activity, which is when the animals usually eat. Ablating AgRP/NPY neurons also disrupts circadian feeding patterns. Although no direct connections from the SCN to AgRP neurons have been identified, their activity correlates with the SCN when mice are feed freely. Sayar-Atasoy et al. (2024) have shown, however, that when feeding is restricted to the light period, AgRP activity is also restricted. Moreover, if food is not forthcoming after the new expected time, activity of AgRP neurons continues to increase, suggesting they figure not just in registering hunger but also expectation of feeding.

There are also inputs to the ARC from PK-2 fibers in the SCN (Méndez-Hernández et al., 2020) as well as projections from AVP neurons in the SCN to kisspeptin neurons in the ARC in female mice. In addition to these direct pathways, there are also indirect routes by which the SCN can affect the control of eating by the ARC. Lesioning all projections between the SCN and the ARC leaves the SCN rhythmic but eliminates circadian rhythms in locomotor activity when mice are in constant darkness (they are maintained if there is a light-dark cycle in the environment; Buijs et al., 2017).^20^ The loss of feeding rhythms may be a consequence of the loss of overall activity rhythms. The influence of the SCN on the ARC can also be mediated by the role of the SCN in modulating PVN activity that in turn affects glucocorticoid release (Méndez-Hernández et al., 2020).

So far there has been less research into the projections from the ARC to the SCN, although Yi et al. (2006) identified projections from the ventromedial ARC to the SCN. Méndez-Hernández et al. (2020) identified projections from AgRP and kisspeptin neurons and showed that the number of projections increases during the night. While the functional role these projections play in modulating the activity of the SCN, their existence, like the existence of receptors for ghrelin and leptin in the SCN itself, suggests that the SCN does receive information about the nutritional state of the animal, which could affect its timekeeping.

The ability of the SCN to respond to zeitgebers other than light^21^ would not diminish its important role as especially relevant for organismal activities such as sleep, eating, and locomotion, and in fact could also provide important information to other tissues, even if they don’t always follow it when they have input from other zeitgebers. But it would suggest that the SCN is more like other clocks, specializing in responding to one zeitgeber but also responsive to other timing information. Embracing such a view, Tahara and Shibata (2018, p. 133) assert, “The difference between the central and peripheral clocks is that the light–dark signal is dominant compared with the feeding signal in the SCN, whereas the feeding signal is dominant in the peripheral clocks.” This suggests adopting a different perspective on the SCN—that it is a part of a large network of circadian clocks that each performs specialized functions but shares its information with others. While still insisting on its primacy by referring to it as the “central nexus,” Starnes and Jones (2023) take a step toward such a view:

The SCN thus exists as a central nexus within a complex, dynamic circadian network that integrates information about the outside world (light intensity) with information about an animal’s internal state (arousal, motivation, hormone levels, etc.). This integrated circadian timing signal is propagated onward to synchronize and coordinate daily rhythms in behavior and physiology.

One can recognize the SCN as a central nexus and of particular importance as it is responsive to a highly reliable zeitgeber, the light-dark cycle, without also embracing that it is at the top of a hierarchy. As we learn more about how different clocks figure in the network of clocks, circadian researchers might come to recognize it as one specialist interacting with others.

## Heterarchy And Local Decision Making

I began in section “Identifying the SCN as *the Clock*” with the early research that identified the SCN as the circadian clock in mammals and in subsequent sections discussed research that revealed clocks distributed among mammalian tissues, how they are networked, and ended with the suggestion that the SCN is just one more clock, one specialized to track information about light. Throughout this history, circadian researchers have privileged the SCN clock, characterizing the overall system as hierarchical. In the introduction, I introduced the term *heterarchy* for systems that deviate from hierarchy in not having a highest-level authority. In this final section, I will further develop the notion of heterarchy, indicate how it deviates from the federated model in one significant respect, and offer a sketch of why evolution is likely to generate a heterarchical-organized circadian control system.

McCulloch (1945) introduced the notion of heterarchy for non-transitive preference orderings, but it can be extended to non-transitive control systems in which controlled mechanisms also exert control over the mechanisms that control them (Pattee, 1991). To explicate this perspective, consider first how mechanisms control other mechanisms. Mechanisms constrain flows of free energy to perform work (Winning and Bechtel, 2018). In many cases, the work performed by a mechanism is to initiate physical activities—digest food or move an organism through its environment. I designate mechanisms that perform these functions *production mechanisms*. Many of the constraints in such mechanisms are fixed during the operation of the mechanism, but some are flexible and can be acted on by other mechanisms. I refer to mechanisms that operate on other mechanisms to change their flexible constraints as *control mechanisms*. Circadian clocks are control mechanisms—they act, for example, on clock-controlled genes, thereby altering the work done by the different tissues of an organism. In the same way that they can control production mechanisms by changing flexible constraints in them, control mechanisms can operate on each other. This raises the question of how control mechanisms are organized.

The immediate relation between a control mechanism and a production mechanism is hierarchical—the control mechanism operates on the controlled mechanism. Those viewing an overall system as hierarchical extend this relation. They also, commonly, assume that multiple production mechanisms respond to the same control mechanism, and that this process is iterated. The result is a pyramid structure, with the top-level controller at the top. Humans commonly attempt to organize social systems—universities, corporations, and the military—in this way. The assumption often is that only such an organization would enable decisions that are made to be successfully implemented.^22^ If multiple controllers acted on the same production mechanism and there was no hierarchical structure that ensures that all controllers function in appropriate ways, one might envisage chaos, much as confronted the railroads before standard time. If someone proposed today to allow individual companies and cities to set their own clocks as they wish, the proposal would be rejected out of hand.

There is, however, compelling evidence that biological control is not hierarchical but heterarchical—individual tissues are affected by multiple control mechanisms and control mechanisms are acted on by multiple other control mechanisms. For example, circadian clocks are just one of many control mechanisms that act on tissues such as the liver. When one mechanism receives conflicting signals from multiple control mechanisms, it, as the recipient, determines what behavior will ensue. Astiz et al. (2019, p. 6) note this important role played by local clocks: “local clocks may primarily be responsible for integrating different timing signals to produce an appropriate response to external stimuli.” In such a situation, the pyramid envisaged for hierarchical systems is inverted and control is heterarchical.

To flesh out the concept of heterarchical organization of control and to show how a system in which control is organized heterarchically might be viable, consider what is commonly thought to be an exemplar of a hierarchical pyramid—the administration of a university. (I will describe a common administrative structure in American universities. Terminology will differ in different countries.) One often sees organizational charts, organized as pyramids, with fewer administrators at each level until there is one individual—president or chancellor—at the top. Faculty are viewed as the production mechanisms, teaching classes and doing research. They are organized into departments that are administered by chairs. Chairs report to deans, and deans to a provost, who reports to the chancellor or president. But if one looks at the actual activities of governance in a university, one often finds that the process is far more heterarchical. Chairs typically do not determine the content of research conducted or courses taught by faculty and typically do not even get to choose which faculty to hire or promote (the faculty or a search committee usually decides). And chairs often do not report to just one higher administrator. In recent decades, the number of administrators has increased as new positions are created with specialized administrative responsibilities. Chairs often end up receiving directives from multiple administrators, which sometimes conflict. The same is often true within departments—faculty may receive input not just from the chair but from different committees or even administrators outside their department. Again, these may conflict. It is faculty or the chair that must determine how to accommodate conflicting input.

At the highest level, the administrative structure may appear to be strictly hierarchical—there is one chancellor or president who is responsible for the university. But often the operative organization is much less hierarchical as different subordinate administrators have to interact with different entities outside the university: funders, civic authorities, accreditation agencies, and so on. Even a micromanaging chief executive is unlikely to be able to oversee all that happens. If one tracks actual administrative activities, not just the published organizational chart, one will identify a complex heterarchical network of interacting administrative entities, not a pyramid.

Administrative structures in social systems like universities were initially designed by humans, and at the outset more closely realize a hierarchical pyramid. Overtime, however, those working within the system implement changes, sometimes to address problems, sometimes in the effort to improve the system, and sometimes just to change things up. Those developing the changes typically do not feel constrained to adhere to the original pyramidal design—they may add a new administrator whose authority overlaps existing administrators and who reports to multiple existing administrators. Sometimes the changes won’t solve the problem or achieve the desired improvement, but if they don’t generate conflicts that cripple the operation of the university, they may still be retained. Institutions like universities are more likely to add administrative positions than to eliminate them. Since the changes that are retained because they have not undermined the operation of the system, the heterarchical organization that results will be one that likely will not cripple the system going forward as the conditions in which it must operate tend to change slowly and resemble those in which the heterarchical organization has functioned adequately.

What I have appealed to generate viable heterarchical control systems in the above example is an analog of natural selection—those variations that enable a university to keep functioning are retained. Biological evolution, however, did not start with a hierarchical system from which it evolved away, but an ancestral organism in which the production and control mechanism sufficed to produce offspring that could themselves reproduce in the environment in which it lived. There likely never was a hierarchical pyramid. Moreover, as Partridge (1982) argues compellingly, biological evolution does not operate by seeking solutions to problems. Variations in organisms that give rise to a lineage of successors will be retained. It is the evolutionary theorist who seeks to explain why variants are retained who identifies a problem that the variation addressed. Importantly, large variants often so disrupt the organism that it cannot survive to reproduction. It may be eliminated quickly. Small variants—bringing a production mechanism under an additional control mechanism or adding a connection between control mechanisms—are less likely to be fatal. If not fatal, the resulting heterarchical organization will be one that may allow for the long-term continuation of the lineage. This is not guaranteed. Organisms die. Lineages of organisms come to an end. Nonetheless, some continue. The diverse forms of life on this planet today provide evidence that variation and selective retention has yielded heterarchical control systems that suffice to maintain organisms.

The heterarchical scheme I have described fits well how the federated account describes peripheral clocks. Clocks in different tissues respond to different zeitgebers and regulate physiological activities both in the same tissue and in others. When one considers other control processes that regulate the activities in various tissues, one encounters an even larger control network. Where it differs with the federated model is with respect to the SCN. Although the SCN is distinctive, it is not above the network of other clocks. Rather, it is a contributor to the heterarchical network. Its control activities are most apparent when other clocks fail, perhaps as a result of not receiving input from its zeitgebers. That, however, does not entail that it is above the other clocks, just different.

Having advanced a theoretical argument that evolution is likely to generate heterarchical control systems, I finish by applying the lesson to the mammalian circadian system and consider its implications for future research. Historically, research on the mammalian circadian system originated with timekeeping by the SCN and only subsequently focused on peripheral clocks. This led investigators to view peripheral clocks from the perspective of the SCN, asking questions such as: Do peripheral clocks maintain the same rhythm as the SCN? and How does the SCN communicate to them? The discovery that some peripheral clocks responded to time of feeding suggested that they were more independent than initially thought. The further discovery that they are interconnected and adjust their timekeeping in response to other peripheral clocks supports reversing the initial hierarchical view. Looking out from individual peripheral clocks reveals that they are situated in a network, receiving inputs both from zeitgebers and other clocks that result in tissue-specific modifications to clock and other genes. In light of the adjustments to clock genes, these local peripheral clocks perform their own timekeeping functions, adjusting their behavior in response to information from other clocks (Woller and Gonze, 2021). The SCN is one component in this heterarchical network—responsive to a very important zeitgeber but also modulating its activity in light of information from other clocks. Going forward, mammalian circadian biology might benefit from setting aside the hierarchical perspective as it may itself be blinding researchers to the richness of the heterarchical interactions between clocks. It may also blind researchers to investigating the distinctive contributions of the different local clocks, both in regulating local activities and in supplying inputs to the heterarchical network of circadian clocks. Abandoning the assumption of hierarchy and identifying relations between circadian clocks may lead to a richer understanding of how the circadian system works.^23^

## Notes

1. The clock shop analogy for the circadian system was first introduced by Herzog and Tosini (2001).

2. Circadian biologists often refer to the suprachiasmatic nucleus (SCN) as the *master* clock and peripheral clocks as *slaves*. As discussed by Berezin (2023), this language was adopted from engineering, perhaps without recognition of its political roots. It is clearly offensive to many people today. Accordingly, I will use terms like *pacemaker* for the SCN and *subordinate* for peripheral clocks while describing the hierarchical perspective.

3. The shell arginine vasopressin (AVP) and core vasoactive intestinal polypeptide (VIP) neurons are only 2 of at least 8 cell types, 5 of which are neurons, in the SCN (Wen et al., 2020).

4. The prominent role the liver has played in research on peripheral clocks reflects its role as a pivotal metabolic organ. It receives both venous and arterial inputs: nutrients, xenobiotics, and pathogenic material from the digestive system arrive at the liver via the portal vein, while from hepatic arteries cytokines, metabolites, hormones, and other endobiotic compounds reach the liver. It carries out a host of biochemical reactions on these molecules, producing many ligands and cofactors as well as serum proteins that protect against pathogenic material. Many of these reactions exhibit a circadian rhythm including metabolism of carbohydrates, lipids, amino acids and bile acid, detoxification, and synthesis of plasma proteins and cholesterol.

5. The preoptic area functions in the regulation of body temperature—inputs from the SCN result in increasing body temperature prior to the expected onset of activity and a reduction at the onset of the resting phase (Liu et al., 2002). One pathway through which the preoptic area communicates to peripheral organs is via the ventral medial hypothalamus (VMH). It sends projections to brown adipose tissue which, by disconnecting oxidative metabolism from ATP synthesis, generates heat. This process is regulated by the circadian clock in the VMH (Orozco-Solis et al., 2016). Temperature is generally a zeitgeber for circadian oscillators (those in the SCN are an exception as the network organization of the SCN renders it temperature insensitive). Peripheral tissues, on the other hand, are sensitive to temperature (Buhr et al., 2010). Accordingly, one way for the SCN to act on other circadian clocks is via altering core body temperature.

6. Oster et al. (2006) offered evidence that the adrenal gland has its own clock that gates the role of adrenocorticotropic hormone (ACTH) in initiating release of glucocorticoids so that more is released at the beginning of the active period (see also Astiz et al., 2019).

7. In a later study, Noguchi et al. (2013) showed that for fibroblasts in very low-density cultures, rhythms were “largely abolished.” But if the cells were co-cultured with wild-type fibroblasts or even ones that were non-rhythmic as a result of deletion of Bmal1, they remained rhythmic. The researchers propose “that diffusible paracrine signals are necessary to generate robust circadian rhythms in single fibroblasts but that these signals do not have to be rhythmic and do not regulate the periods of fibroblasts” (p. 184).

8. Reflecting the concern with the health consequences presented by that abnormal zeitgebers, such as eating at night in humans, Woller and Gonze (2021) refer to disrupted circadian clocks as “twisted clocks.” While the negative health effects of twisting circadian clocks are certainly important, we should also recognize that there are circumstances in which it is adaptive to twist one’s clocks.

9. Focusing not on clock genes, but the products of genes that figure in metabolism, Manella et al. (2021) found that transcription of many genes that coded for proteins in glucose and glycogen metabolism that are rhythmic in the liver with normal feeding ceased to be rhythmic with altered time-restricted feeding. Since these proteins were no longer oscillating in anticipation of food intake, blood glucose ceased to be held constant and itself oscillated with feeding.

10. Tahara et al. (2012) explored what happened if, after training an animal by restricting feeding to an abnormal time, it was again allowed to feed ad libitum. They found that the altered phase of activity in the liver persisted, but the amplitude of the oscillation was significantly dampened.

11. An anonymous reviewer raised the concern that forced feeding in a laboratory situation does not reflect the situation in natural settings in which food cues only become effective when the animal also adjusts its sleep-wake cycle, which is under SCN control. Acknowledging that a zeitgeber becomes effective only in the context of other circadian adjustments in natural settings does not, though, make food availability itself any less of a zeitgeber for peripheral clocks.

12. Similar results showing different effects of daytime feeding on different tissues had been found previously by Bray et al. (2013) and de Goede et al. (2018).

13. Many rhythmic functions associated with the liver are carried out by different cell types. Guan et al. (2020) showed that altering rhythms in hepatocytes altered rhythms in other liver cell types, affecting the adjustment of other liver cells to time-restricted feeding.

14. Koronowski et al. also found that if these mice with a functioning clock only in the liver were maintained in an environment with a light-dark cycle, rhythms affecting glucose metabolism were restored. However, in mammals only the eyes are responsive to light. So somehow in Koronowski et al.’s study, the liver was receiving a zeitgeber signal from the eyes. The normal route would be through the entrainment of the SCN, but the clock in the SCN was disabled. Based on research employing a similar protocol, Husse et al. (2014) propose a pathway through the SCN that is maintained even when SCN clock function is impaired.

15. In several cell types, including neurons, insulin (as well as insulin-like growth factor 1, IGF-1) has been shown to act on *Per1* and *Per2* expression, mediated by the mechanistic target of rapamycin (mTOR) pathway (Crosby et al., 2019).

16. In a subsequent study focused on protein levels (measured at one timepoint), the same research group (Smith et al., 2023b) found a greater effect for the restored clock than for restriction of feeding. Of those proteins that were altered in mice without a clock, about half of those in the liver were rescued by restoring the clock in the liver and muscle as well as about a quarter of those in muscle. Restricting feeding had little effect.

17. Research by Bookout et al. (2013) suggests an alternative route involving fibroblast growth factor 21 and the pathway on which it acts, the FGF21-β-Klotho pathway. Fasting results in increased FGF21 secretion by the liver, which acts on the β-Klotho pathway to cause a switch to oxidative metabolism in peripheral tissues. Through receptors on the SCN, it acts to reduce reproductive and locomotor activities.

18. Since both ghrelin expression and Per1/Per2 expression in oxyntic cells in the stomach are rhythmic and responsive to feeding time, LeSauter et al. (2009) propose that these cells might be the food-entrainable oscillator. This is further supported by the fact that ghrelin, at least in humans, has an E-box to which Per1 and Per2 can bind.

19. A recently discovered further inhibitor is liver-expressed antimicrobial peptide 2 (LEAP2), which is secreted by the liver, kidney, stomach, duodenum, and jejunum in the fed state and acts as an antagonist on growth hormone secretagogue receptors (GHSRs).

20. In Buijs et al.’s study, circadian oscillations in core body temperature were also eliminated when in constant darkness with the disconnection of the SCN from the ARC. The medial preoptic area receives inputs from both the ARC and the SCN (Guzmán-Ruiz et al., 2015). There is evidence that the role of the SCN in regulating temperature is affected by fasting that may be mediated by the ARC. The temperature decline at the beginning of the light phase is greater in fasted animals, and this decline is eliminated when the SCN is lesioned (Liu et al., 2002)

21. There are other non-photic inputs to the SCN, both synaptic and hormonal, than those discussed here. For example, the ventral SCN has serotonin receptors that respond to serotonin from neurons originating in the median raphe nuclei, likely conveying arousal information and feedback from locomotor activity. Starnes and Jones (2023) report that about 40 brain regions project monosynaptically to the SCN. Many of these project to VIP neurons or to a distinct population of cholecystokinin (CCK) synthesizing neurons (Yuan et al., 2018). CCK expressing neurons have projections to both AVP and VIP neurons and may affect their resetting in response to non-photic stimuli. In addition, dopamine receptors have been found in the SCN and there is evidence that they receive input from the ventral tegmental area (Grippo et al., 2017).

22. Such an organization imposes an enormous epistemic challenge—the highest-level controller must have access to all the information needed to make good choices and understand how to implement activities throughout the hierarchy to implement the choices. This may be why many social institutions organized hierarchically turn out to fail.

23. I would be remiss if I didn’t acknowledge a challenge presented by heterarchical networks. It is relatively straightforward to track activities within a hierarchical pyramid. Complex dynamical interactions are minimized. It is much harder to track activities in a complicated heterarchical network in which there may be dynamical activities operating on different timescales. To understand what will happen in such networks, researchers will likely need to invoke computational models, much like those that have been deployed to understand dynamical behavior in the network in the SCN (Abel et al., 2016; Relógio et al., 2011; Bechtel, 2016), analyzing them using conceptual tools developed for oscillatory dynamical systems (Abel and Doyle, 2016).
